# Formation and Evolution of sp^2^ Hybrid Conjugate Structure of Polyacrylonitrile Precursor during Stabilization

**DOI:** 10.3390/ma15010030

**Published:** 2021-12-21

**Authors:** Ruihao Dong, Jianglu Wu, Ting You, Weiyu Cao

**Affiliations:** 1 State Key Laboratory of Organic-Inorganic Composites, Beijing University of Chemical Technology, Beijing 100029, China; 2020210313@buct.edu.cn (R.D.); wujl1130@126.com (J.W.); yout0123@126.com (T.Y.); 2 The Key Laboratory of Education Ministry on Carbon Fiber and Functional Polymer, Beijing University of Chemical Technology, Beijing 100029, China

**Keywords:** polyacrylonitrile precursor, thermal stabilization, sp^2^ hybrid, conjugated structure

## Abstract

The generated sp^2^ hybrid conjugate structure of a C atom, which resulted from the chemical reaction affected by temperature and time, is an effective six-membered ring planar structure of the final carbon fiber. This kind of hybrid conjugate structure determined the formation of the final structure and mechanical properties of carbon fiber. In this paper, the formation and evolution of sp^2^ hybrid conjugated structures of PAN precursor during thermal stabilization were investigated by Raman, UV-vis and ^13^C-NMR methods. The results indicated that with the increase of stabilization temperature, the degree of the sp^2^ hybrid conjugated structure of stabilized PAN fiber increases “linearly”, while the content of the sp^2^ hybrid carbon atoms increases with “S-type”. The final sp^2^ hybrid conjugated ring structure is mainly composed of single-ring, double-ring, triple-ring, and double-bond structures. Compared with the time factor, the temperature effect plays a decisive role in the formation of the sp^2^ hybrid conjugate structure.

## 1. Introduction

PAN-based carbon fiber has applied in various fields due to its unique properties such as high ratio strength and modulus. In order to further meet the demand for the high performance of carbon fiber, many contributions have been done in the modification of spinning solution, which include doping high-performance materials in spinning liquid or choose a new solvent. In this case, chemical and structural transformations occur already at the stage of fiber production [1,2,3,4]. However, thermal stabilization is another key process of the preparation for polyacrylonitrile (PAN)-based carbon fibers, in which PAN linear molecular chains are transformed into a heat-resistant ladder structure through chemical reactions. Since it is an initial stage of the organic–inorganic structural transition, thermal stabilization has had an important influence on the properties of final carbon fibers [5,6,7,8]. In the process of thermal stabilization for PAN fibers, a lot of chemical reactions such as cross-linking, oxidation, cyclization, and dehydrogenation occurred. In this research field, a lot of work was reported previously. It is believed that the cyan group (-C≡N) was involved in the most reactions above [9,10,11,12,13,14]. In addition, according to the results of IR and UV-vis, Zhou speculated that the cross-linking and fracture reaction of -C=N-N=C- would occur during the stabilization process of PAN fibers, while oxygen could retard the generation of cross-linking structure [15]. Liu studied the effect of temperature on the diffusion of oxygen along the radial direction of PAN fibers during stabilization. It was believed that the generation of a “skin-core” heterogeneous structure was related to the too highly stabilized temperature [16]. Sivy reported that when the acrylonitrile and acrylamide copolymers were treated at a relatively low temperature in an oxygen-containing atmosphere, oxygen participated in the chemical reactions and the structure of 1,1,2-trisubstituted alkenes was formed [17]. Lei believed that unsuitable cyclization degree and oxygen content were the main reasons for the formation of the defective structure as well as for the density of final carbon fiber [18]. 

Actually, during the thermal stabilization process, carbon atoms are expected to convert to sp^2^ hybrid conjugate ring structures, which is the basic unit structure of the pseudo-graphite layer in the final carbon fibers. This is the only path for the conversion from conjugate structures, which consists of an aromatic ring and an unsaturated double bond, to the larger scale pseudo-graphite layer structure [9,10,11,12]. However, more attention was paid to the generation of new chemical groups through Infrared spectra or other methods to trace the transformation of fiber structure during stabilization in previous studies. The formation and evolution of a significant sp^2^ hybrid conjugated ring structure during stabilization, which can be inherited by the final carbon fiber, was not clarified in this stage. As a result, the structural relationship between the thermal-stabilized fiber and the final carbon fiber was not established. Therefore, it is of great significance to study the formation of the sp^2^ hybrid conjugated structure in the process of thermal stabilization for obtaining high-performance carbon fibers.

From the viewpoint of the reaction activation energy and the kinetics of stabilization, the sequence and extent of various chemical reactions will lead one to the formation of multifarious chemical structures in PAN-stabilized fibers. Therefore, temperature and time effect play a crucial role in the formation of the structures in PAN fibers. In this paper, the effects of temperature and time on the formation and evolution of sp^2^ hybrid conjugated structures of PAN thermal-stabilized fibers were studied by Raman spectroscopy. The crosslinking of this conjugated ring structure, that is, the formation of the skeleton of the ladder structure, was traced by UV-visible absorption spectroscopy (UV-vis) and nuclear magnetic spectroscopy (^13^C-NMR).

## 2. Materials and Methods

### 2.1. Preparation of PAN Thermal-Stabilized Fibers

PAN precursor was prepared by wet-spinning from PAN/DMSO solution, which was obtained by the copolymerization of acrylonitrile and itaconic acid monomers using 2,2-azobisisobutyronitrile (AIBN) as initiator at 62 °C. The content of itaconic was 0.5 mol%. The concentration of the spinning solution was 20 wt%, and the number average molecule weight of PAN was about 80,000. The prepared spinning solution (60 °C) was extruded through a 3000 holes spinneret into the coagulation bath composed of DMSO and water solution with certain concentration and temperature followed by washing, drying, and stretching. The diameter of PAN monofilaments was about 14 μm. 

To get PAN-stabilized fiber samples at different treatment temperatures, the precursor was heat treated every 10 °C in the range of 180 to 280 °C, respectively, for 7.5 min in the air condition continuously.

PAN thermal-stabilized fibers treated at different times were obtained by adjusting the speed of the roller to make the treatment time as 2.5, 5, 7.5, 10, and 12.5 min at the same temperature in air condition.

### 2.2. Characterizations

Raman spectra of the samples were measured by a InVia-RM2000 Microscopic Confocal Raman Spectrometer, Renishaw, U.K. The laser wavelength and power were selected as 785/532 nm and 0.05 mW, respectively. Scanning range of Raman shift was set from 800 cm^−1^ to 2300 cm^−1^, with DM2500M type 50× objective. The exposure duration and cycle number were 100 s and 3 times, respectively. 

^13^C-NMR spectra were collected by AV-300, made by Bruker company, Bremen, Germany. The resonance frequency and rotation frequency were selected as 73.5 MHZ and 12 kHz, respectively. The pulse width was set as 6.6 μS, and 10,000-times scanning was accumulated by applying the CP/MAS probe with 4 mm.

The UV-vis absorption spectra were measured by integrating sphere method at Shimadzu UV-2450, Japan. The wavelength range was set from 190 to 800 nm. The scanning interval was 0.1 nm, and the slit width was 5 nm. 

## 3. Results and Discussion

### 3.1. The Effect of the Temperature on the Formation and Evolution of sp^2^ Hybrid Conjugate Structures for PAN Fibers

#### 3.1.1. Raman Spectra Analysis

**Figure 1:**
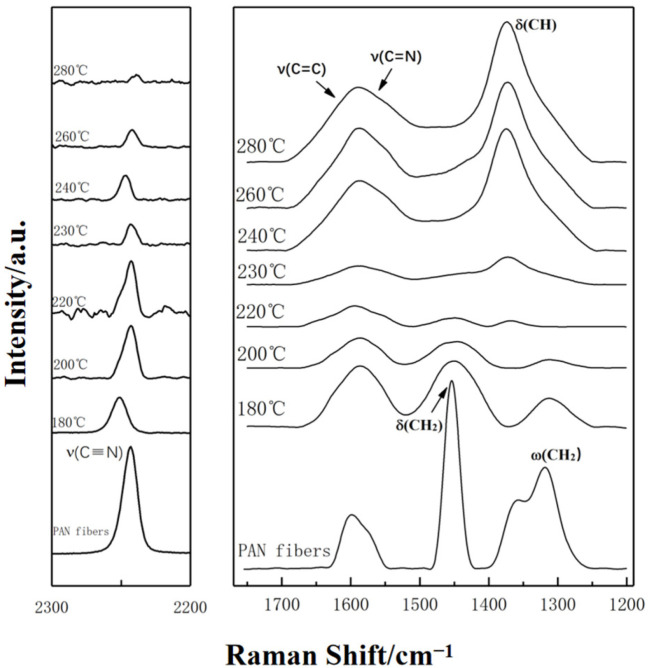
Raman spectra of PAN fibers treated at different thermal stabilization temperatures.

In-situ measurement results of Raman spectra of PAN fibers treated at the temperature from 180 to 280 °C in air are shown in Figure 1. It is clear that the characteristic peak at 2240 cm^−1^, which represents the stretching vibration of cyan group [ν(-C≡N)], was decreased continuously as the temperature increased. The variety of the peaks representing the formation vibration δ(CH_2_) at 1455cm^−1^ and wagging vibration ω(CH_2_) at 1320 cm^−1^ for methylene had the same regularity as ν(-C≡N). There were two types of stretching vibration mode at 1585–1600 cm^−1^: the characteristic shoulder peaks of ν(-C=N-) and ν(-C=C-) [19,20,21,22]. The absorbance of these bands gradually increased with increasing temperature. At 220 °C, it could be observed that the characteristic peak of δ(CH_2_) almost completely disappeared, while a new characteristic peak representing the formation vibration δ(CH) of hypo-methyl (CH) appeared at 1360 cm^−1^, which gradually increased as the temperature increased. These phenomena suggested that the conversion of cyan group (-C≡N) to cyanide(-C=N) started when the temperature was 180 °C. When the temperature was increased up to 220 °C, dehydrogenation reaction occurred and the structure of -C=C- was formed. When the temperature reached 240 °C, cyclization occurred more rapidly, and a large number of cyan were converted to cyanides.

**Figure 2:**
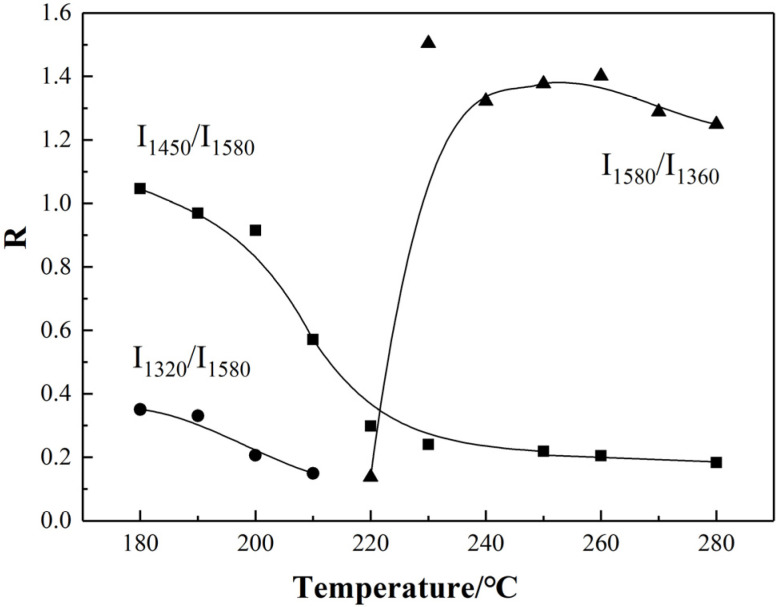
Variation of characteristic structure for PAN thermal-stabilized fibers with temperature.

After being stabilized at different temperatures, the original structure of PAN fibers was developed because of the chemical reactions of cross-linking, oxidation, cyclization, and dehydrogenation. The variation tendency of relative intensity ratio for different characteristic peaks with temperature are shown in Figure 2. It can be observed that I_1450_/I_1580_ (I_δ(CH_2___)/_I_ν(C=N)_) and I_1320_/I_1580_ (I_ω__(CH_2___)/_I_ν(C=N)_) decreased with increasing temperature, while the I_1580_/I_1360_ (I_ν(C=N)_/I_δ(CH_2___)_) increased at first and then decreased slightly. Therefore, the gradually increase of I_1580_ (C=N) could be due to the transformation from -C≡N to -C=N during stabilization. At the initial stage of thermal stabilization from 180 °C to 210 °C, the absorbance of characteristic peaks that, respectively, represented the ω(CH_2_) at 1320 cm^−1^ and the δ(CH_2_) at 1450 cm^−1^ of PAN fibers was decreasing gradually. After 220 °C, the characteristic peak of δ(CH) at 1360 cm^−1^ appeared, which represented structural transformation from CH_2_ to CH due to the start of dehydrogenation. On the other hand, I_1585_/I_1360_ increased first and then decreased, indicating that the cyclization was a domain, compared to dehydrogenation at 220–230 °C, and both tended to be stable at 240–280 °C.

The degree of sp^2^ hybrid conjugation for stabilized PAN fibers was characterized by its conjugated structure composition. It was defined here that the relative ratio of I_C=N_ and I_C≡N_ in Raman spectra was applied to characterize the transition from -C≡N to -C=N quantitively [22].

(1) $$\mathrm{Degree}\;\mathrm{of}\;\mathrm{conjugation}=\frac{I_{1580{\mathrm{cm}}^{-1}}}{I_{1580{\mathrm{cm}}^{-1}}+A\times I_{2240{\mathrm{cm}}^{-1}}}\times 100\%$$

In this equation, $I_{1580{\mathrm{cm}}^{-1}}$ represented the I_C=N_ in Raman spectra, $A\times I_{2240{\mathrm{cm}}^{-1}}$ represented the I_C≡N_, and the A represented the correction coefficient.

**Figure 3:**
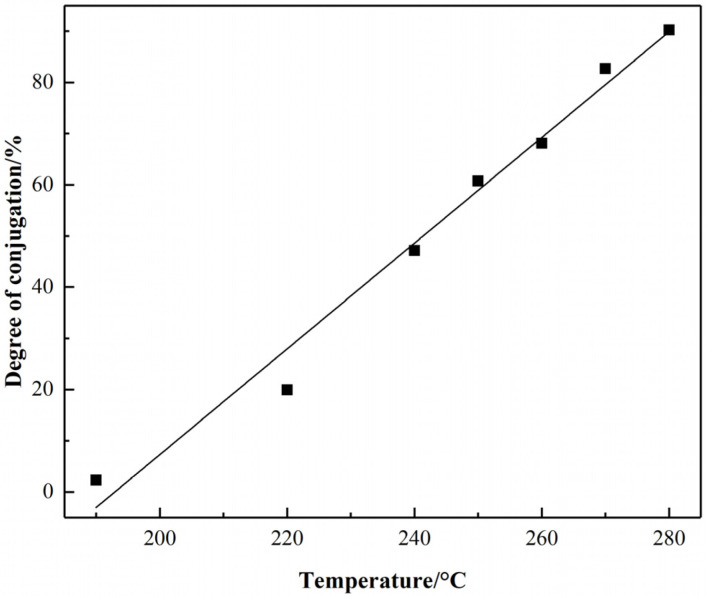
sp^2^ hybrid conjugation degree for PAN fiber with the increasing temperature.

The variation tendency of conjugation degree for PAN fiber with the temperature in the thermal stabilization was shown in Figure 3. It can be observed that the degree of sp^2^ hybrid conjugation structure for PAN pre-oxidized fiber grown in a “linear” trend approximately with the increase of temperature, which indicated that the formation rate of hybrid conjugated structures, was almost uniform with the increase of temperature during the stabilization process.

#### 3.1.2. UV-Vis Absorption Spectra Analysis

The sp^2^ hybrid conjugated structures of stabilized PAN fibers were traced by UV-vis method. In the UV-vis spectra, the peak intensity depends on the amount of conjugation structures while the red-shifting can be used to characterize the change of conjugation structure dimension [23,24,25,26,27]. The absorption wavelengths in the range of 190 to 800 nm are most likely due to the π-π* and n-π* electron transitions. The maximum absorption wavelength (λ_max_) is related to the dimension of sp^2^ conjugated ring structure. For example, the UV-vis absorption bands of polycyclic aromatic hydrocarbons were located at 204 nm for benzene, 275 nm for naphthalene, 374 nm for anthracene, etc., respectively [26,27]. The relationship between the characteristic structures such as benzene, pyridine, and phthalazine and corresponding absorption bands was also established [26,27]. Therefore, it is appropriate to characterize the dimension of ring conjugation for thermal-stabilized fibers by the spectral band of UV-vis spectra. 

**Figure 4:**
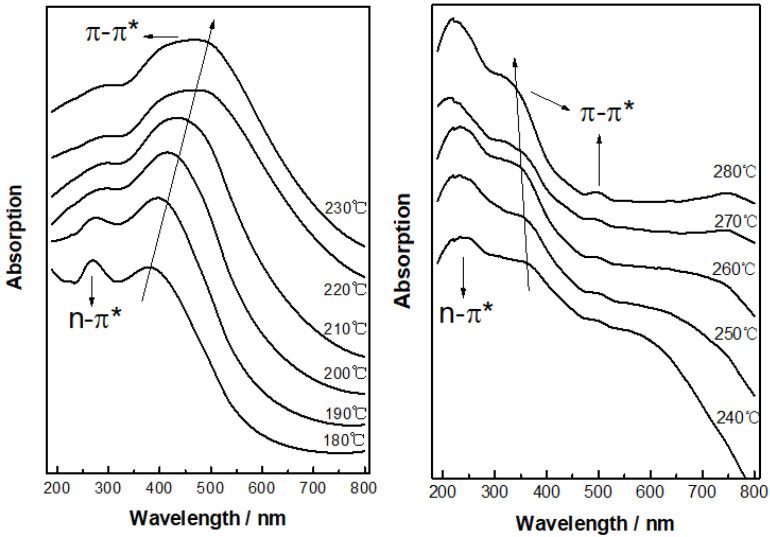
UV-vis spectra of PAN fibers treated at different thermal stabilization temperatures.

UV-vis spectra of PAN fibers treated at different thermal stabilization temperatures are shown in Figure 4. It can be seen that the absorption peaks near 270 nm and 380 nm appeared on the UV-vis absorption curves at 180 °C. This could be due to the strong π-π* absorption and the weak n-π* absorption of two conjugated structures with different conjugation degrees [23,24]. The variety of intensity for the two peaks was also studied in different temperatures by Lei [25]. Similarly, in present work, with the increase of stabilization temperature, the absorption peak at 270 nm broadened gradually and slightly red-shifted by about 28 nm. However, the absorption peak at 377 nm shifted gradually in the direction of larger wavelength with the increase of temperature. The peak position had an obvious red-shifting tendency up to around 481 nm. The red-shifting of UV absorption bands represented that the increasing of the conjugated degree resulted from the increasing of delocalization of π-electron in sp^2^ conjugation. Therefore, it meant that the degree of sp^2^ hybrid conjugated structures increased from 180~230 °C. When the thermal stabilization temperature was between 230 and 280 °C, there were three different types of conjugated structures in stabilized PAN fibers, of which λ_max_ was located near 240 nm, 350 nm and 490 nm, respectively. It can also be noticed that the absorption peaks at 240 nm and 350 nm had slight blue-shift on the whole, which resulted in the decreasing of the conjugated degree for stabilized PAN fibers.

**Figure 5:**
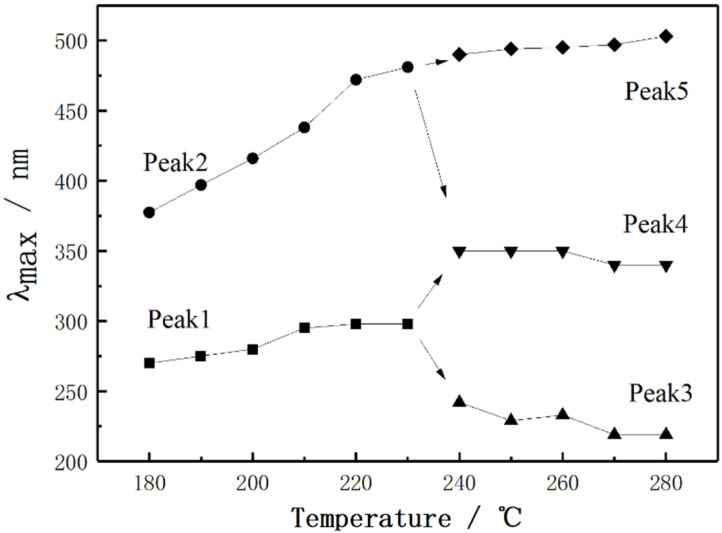
Variation of the λ_max_ position corresponding to the UV-vis absorption spectrum of PAN fibers during stabilization process.

The position of λ_max_ corresponding to the UV-vis absorption spectra of PAN fibers with the change of temperature during stabilization is described in Figure 5. It can be seen that λ_max_ increased as the temperature increased in the range of 180–230 °C. When the thermal stabilization temperature was in the range 230–280 °C, there were three different types of conjugated structures deduced from the UV-vis spectra and almost no change with the increase of temperature. Based on the relationship between the location of absorption bands and the dimension of conjugate rings [26], as well as the analysis above, the influence of temperature on the evolution of sp^2^ hybrid conjugated structure for PAN fiber can be concluded. Firstly, in the range of 180–230 °C, the conjugated structure represented by λ_max_ near 270 nm was changed from a single ring structure with two double bonds to a two-ring structure. That is, the crosslinked structure of two molecular chains was transformed to a complete ring structure. The conjugated structure represented by λ_max_ near 370 nm changed from two conjugated rings with two double bonds to a triple-ring structure. Secondly, in the range of 230–280 °C, a double-bond structure and a two-ring structure with a double bond were generated because of the dissociation and transformation of the two-ring structure. Meanwhile, the triple-ring structure transformed to a double ring with a double bond and a triple ring with a double bond.

#### 3.1.3. ^13^C-NMR Analysis

**Figure 6:**
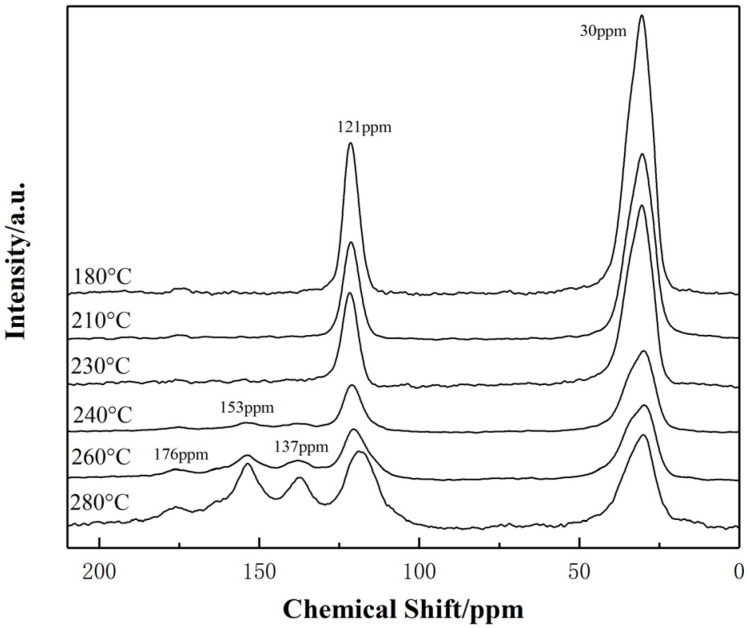
^13^C-NMR spectra of PAN fibers stabilized at 180–280 °C.

^13^C-NMR was used to analyze the types of carbon atoms and the change of content for sp^2^ hybrid carbon atom during stabilization process of PAN fibers [21]. The ^13^C-NMR spectra of PAN fibers with different temperatures during stabilization process are shown in Figure 6. At a temperature of 180 °C, only characteristic peaks at 30 ppm (-CH_2_) and 121 ppm (-C≡N) appear in the NMR spectra since the cyclization and dehydrogenation of PAN fibers have not yet begun. When the temperature was increased from 180 °C to 260 °C, characteristic peaks near 176 ppm (-C=O) and 153 ppm (-C=N-) appeared in the NMR spectra successively, indicating that the cyclization reaction had been started simultaneously with the oxidation reaction. When the temperature was increased from 240 °C to 280 °C, a characteristic peak at 137 ppm appeared, indicating the generation of -C=CH- group due to the beginning of dehydrogenation reaction. At this time, cyclization, dehydrogenation, and oxidation reactions, which were all the characteristic reactions to generate sp^2^ hybrid carbon atoms, occurred simultaneously. Generally, sp^2^ unsaturated carbon atom was more likely to be produced by dehydrogenation, which could be accelerated after 240 °C especially. When the temperature reached 260 °C, the cyclization tended to be moderate and the dehydrogenation and oxidation were sustained. The characteristic peaks at 137 ppm (-C=CH-), 153 ppm (-C=N-), and 176 ppm (-C=O) became more obvious, which indicated that the proportion of characteristic structures in PAN-stabilized fibers increased.

The parameter *C_sp_*_2_% was defined here to characterize the relative content of sp^2^ carbon atom, as below:

(2) $$C_{sp^{2}}=\frac{A_{C(sp^{2})}}{A_{c}}\times 100\%$$

In this equation, $A_{C(sp^{2})}$ represented the content of *C* atoms with sp^2^ hybrid structure based on the NMR of PAN-stabilized fibers. There were mainly three kinds of structure for C atoms, namely, -C=C-, -C=N-, and -C=O. $A_{C}$ represented the content of all forms of C atoms in the NMR spectra of PAN-stabilized fibers.

**Figure 7:**
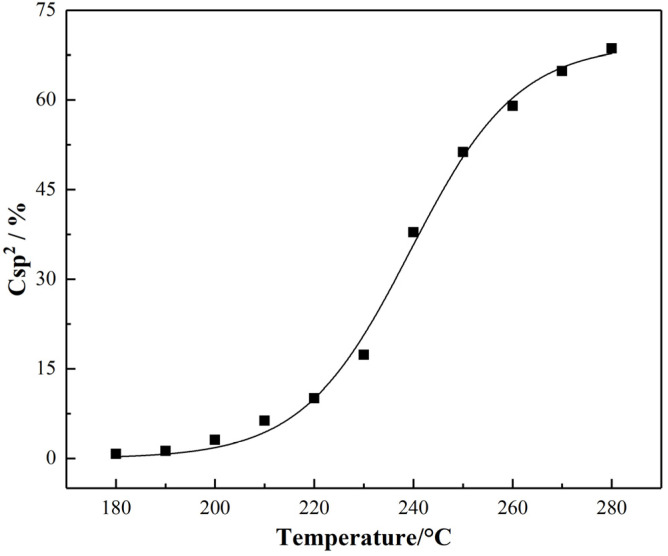
Variety of relative content of sp^2^ hybrid carbon atoms of PAN fibers during stabilization.

The content of sp^2^ hybrid carbon atoms for PAN-stabilized fibers grown in an “S-shape” with the increase of temperature can be seen in Figure 7. At 180 °C, there was a low content of sp^2^ hybrid carbon atom. In the range of 180–220 °C, the content of sp^2^ hybrid carbon atoms increased slowly. At this time, the main reaction was the cyclization of linear PAN, and the -C≡N structure was transformed to -C=N. In the range of 220–250 °C, the content of sp^2^ hybrid carbon atoms grew rapidly. In this case, the dehydrogenation of cyclized PAN mainly occurred while a small part of linear PAN occurred dehydrogenation. The CH_2_ structure was transformed into -CH, and the -C=C- structure was generated. Linear PAN molecular chains that do not cause dehydrogenation are also oxidized after 240 °C, resulting in the generation of -C=C- and -C=O structures. In the range of 260–280 °C, the chemical reactions began to slow down and eventually became stable.

### 3.2. The Effect of Time on the Formation and Evolution of sp^2^ Hybrid Conjugate Structure for PAN Fibers

#### 3.2.1. The Characterization of sp^2^ Hybrid Conjugated Structure for PAN-Stabilized Fibers by Raman Spectrum

**Figure 8:**
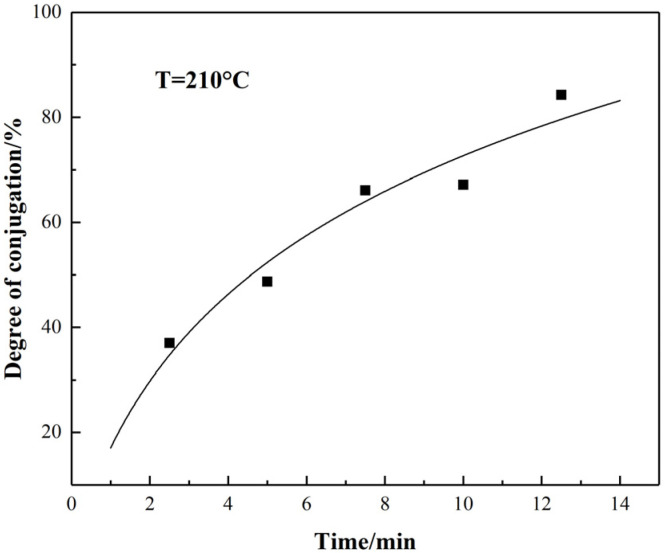
Relationship between the degree of sp^2^ hybrid conjugation and time for stabilized PAN fiber.

The relationship between the degree of sp^2^ hybrid conjugate for stabilized fiber with time is shown in Figure 8. Under a series of stabilization temperature conditions, the conjugated degree of PAN-stabilized fibers increased as the time increased in the range of 2.5–12.5 min. The increasing rate was relatively fast in the early 7.5 min stage of stabilization, while it decreased when the time was further extended. This indicated that the extending of reaction time could promote the formation of sp^2^ hybrid conjugate and carbonyl structure with a certain limitation since the cyclization, dehydrogenation, oxidation, and crosslinking could be more complete kinetically.

#### 3.2.2. UV-vis Spectra Analysis 

**Figure 9:**
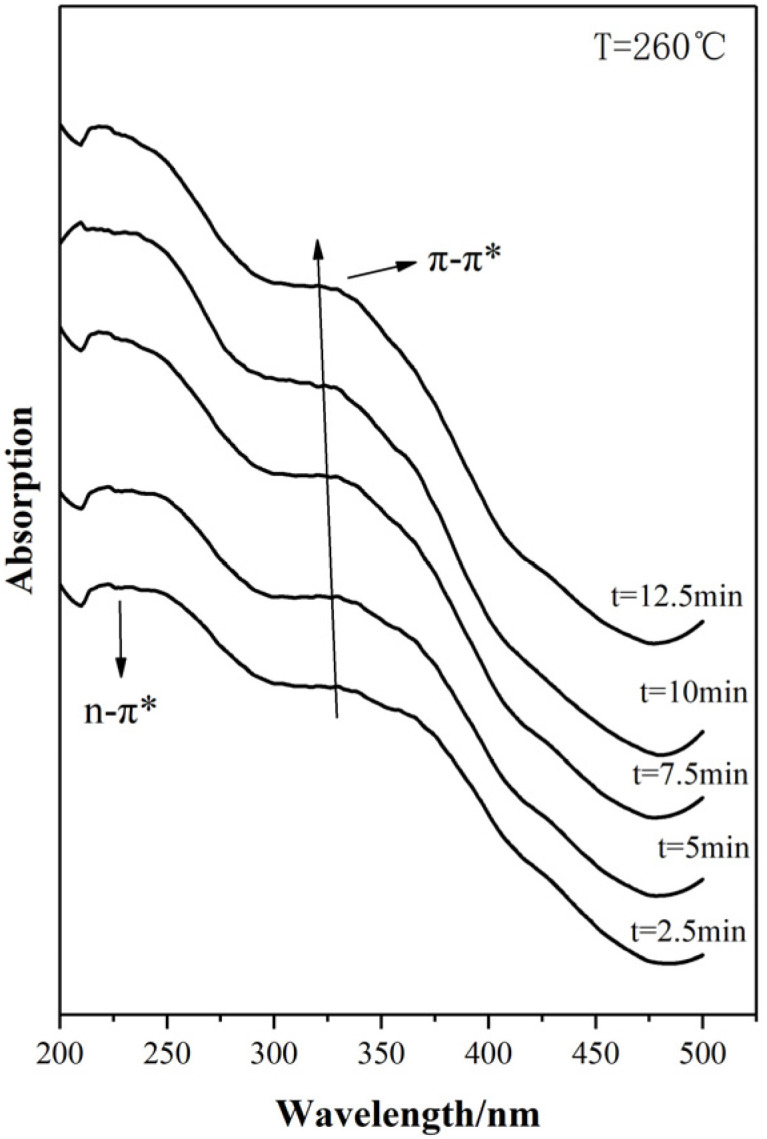
UV-vis absorption spectra of stabilized PAN fibers with different treatment times.

UV-vis absorption spectra of PAN fibers stabilized with different heat-treatment time at 260 °C is shown in Figure 9. With the increase of heat-treatment time, the UV-vis absorption for PAN-stabilized fibers was increased while there is almost no shifting for the maximum wavelength (λ_max_). It can be concluded that with the increase of heat-treatment time, the amount of conjugated ring structure was increased while the dimension of conjugated ring was kept.

## 4. Conclusions

The formation and evolution of the sp^2^ hybrid conjugate carbon structure was traced by Raman spectra combined with UV-vis and NMR. The results indicated that:

1. The degree of the sp^2^ hybrid conjugate for PAN-stabilized fibers increased “linearly” approximately with the increase of stabilization temperature from the Raman spectra analysis. It could also be increased gradually with the extension of heat-treatment time. As the results from ^13^C-NMR show, the amount of sp^2^ hybrid carbon atoms increased in a “S-type” tendency with the increase of stabilized temperature.
2. When the thermal stabilization temperature was in the range of 180–230 °C, the conjugated structure of PAN-stabilized fibers changed from a single ring structure plus two double-bond structures to a conjugated double-ring structure, two conjugated rings, and two double-bond changed to triple-ring conjugated structures. In the temperature range of 230–280 °C, the dissociation and transformation of the double-ring structure and triple-ring structure occurred, resulting in the formation of three types of conjugated structures at same time. These were the single-ring and double-bond, double-ring and double-bond, triple-ring and double-bond structures, respectively.
3. With the extension of thermal stabilization time, the amount of conjugated ring structures increased, but the dimension of conjugated ring structures did not change. Therefore, the dimension of conjugated ring structures mainly depended on the stabilized temperature. Stabilization time did not play an important role in this.

## Data Availability

Data contain within the article.
